# Distinctive magnetic resonance imaging features of progressive supranuclear palsy with cerebellar phenotype: hummingbird and Mickey Mouse signs

**DOI:** 10.11604/pamj.2026.53.114.50280

**Published:** 2026-03-04

**Authors:** Sahil Chaudhari, Pallavi Harjpal

**Affiliations:** 1 Department of Neuro-physiotherapy, Ravi Nair Physiotherapy College, Datta Meghe Institute of Higher Education and Research, Sawangi, Meghe, Wardha, Maharashtra, India

**Keywords:** Progressive supranuclear palsy, axial rigidity, intentional tremors

## Image in medicine

A patient in their 60s presented with a progressive history over two years of unsteadiness of gait, frequent falls, impaired coordination, and slurred speech. Neurological examination revealed axial rigidity, bradykinesia, intentional tremors of the upper limbs, wide-based ataxic gait, limb dysmetria, impaired rapid alternating movements, and increased tone with cogwheel rigidity. Vertical gaze palsy consistent with supranuclear ophthalmoplegia was noted. These clinical features are indicative of progressive supranuclear palsy (PSP) with cerebellar involvement. The magnetic resonance imaging findings revealed, in the sagittal T1-weighted sequence (A: red arrow), marked atrophy of the midbrain tegmentum with relative preservation of the pons, producing the classical “hummingbird sign” (also known as the “penguin sign”), an established neuro-imaging hallmark supportive of PSP diagnosis. Significant atrophy of the cerebellum, particularly affecting the vermis and hemispheres, contributes to the enlargement of the fourth ventricle. The axial T2-weighted sequence (B: red circle) at the midbrain level shows characteristic atrophy with reduction in the anteroposterior diameter of the midbrain tegmentum, consistent with the “Mickey Mouse sign”, another well-recognised radiologic marker supportive of PSP. Cerebellar atrophy is evident as volume loss in the cerebellar hemispheres, visible on these axial slices. Together, these clinical and imaging findings confirm the diagnosis of the cerebellar phenotype of PSP (PSP-C). Recognition of this variant is essential for guiding management, which primarily involves supportive care through physiotherapy and occupational therapy, as pharmacological treatment responses are typically limited. Early diagnosis facilitates fall prevention strategies, tailored rehabilitation, and patient and caregiver counselling regarding disease progression.

**Figure 1:**
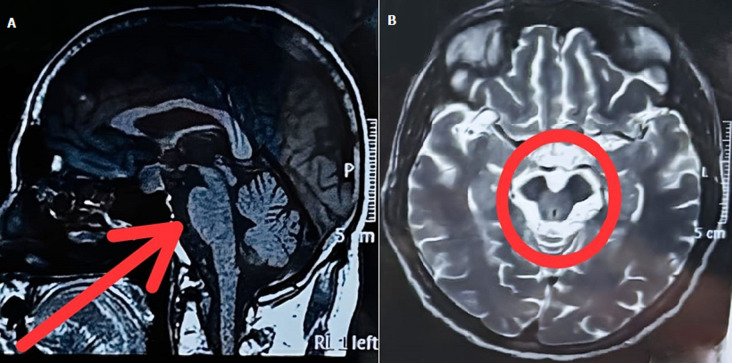
A, B) sagittal T1-weighted (red arrow) and axial T2-weighted (red circle)

